# Impact of functionality and grading on survival in pancreatic neuroendocrine tumor patients receiving peptide receptor radionuclide therapy

**DOI:** 10.3389/fendo.2025.1526470

**Published:** 2025-04-15

**Authors:** Annie Mathew, David Kersting, Wolfgang P. Fendler, Johanna Braegelmann, Dagmar Fuhrer, Harald Lahner

**Affiliations:** ^1^ Department of Endocrinology, Diabetes and Metabolism and Division of Laboratory Research, University Hospital Essen, University Duisburg-Essen, Essen, Germany; ^2^ Department of Nuclear Medicine, University Hospital Essen, University of Duisburg-Essen, Essen, Germany

**Keywords:** pancreas, survival, PRRT, neuroendocrine tumor, PanNET, functionality

## Abstract

**Background:**

Peptide receptor radionuclide therapy (PRRT) is a well-established treatment option for neuroendocrine tumors (NET), yet randomized controlled trials have not provided data on its impact on overall survival. The real-world efficacy of PRRT and its association with tumor functionality and grading in pancreatic neuroendocrine tumors (PanNET) remains underexplored.

**Methods:**

A retrospective analysis of 166 patients with histologically confirmed metastatic PanNET was performed. Subgroup analyses examined progression-free survival (PFS) and overall survival (OS) following PRRT cycles, stratified by tumor grading, tumor functionality and bone metastases.

**Results:**

Of 166 patients, 100 (60.2%) received PRRT with a median of four cycles. In the PRRT cohort, 68% of patients had deceased. PFS after four and eight consecutive cycles was 20 and 18 months, respectively (p=0.4). OS for the entire cohort was 79 months, with patients receiving 4+ cycles of PRRT having an OS of 87 months and those receiving 5+ cycles achieving an OS of 100 months. Patients with grade 1 or 2 tumors had a significantly longer median OS of 97 months compared to 74.5 months for grade 3 tumors (p = 0.0055). There was no significant difference in OS between functioning and non-functioning tumors after PRRT. Patients with bone metastases who received PRRT had a significantly shorter OS than those without (74 vs. 89 months, p = 0.013). In 19% of patients who received PRRT, therapy was discontinued due to progressive disease, toxicity or death.

**Conclusions:**

Patients receiving extended cycles of PRRT showed improved survival outcomes in metastatic PanNET, particularly in patients with lower tumor grades and without bone metastases. No survival difference was seen between functioning and non-functioning PanNET, while patients with grade 3 tumors and bone metastases had significantly shorter survival despite PRRT.

## Introduction

Peptide receptor radionuclide therapy (PRRT) is an effective treatment for advanced neuroendocrine tumors (NET) of the gastrointestinal (GI) tract, but there is limited evidence regarding its efficacy in patients with pancreatic NET (PanNET) (1–3).

Current European Neuroendocrine Tumor Society (ENETS) guidelines recommend PRRT for patients with metastatic grade 1 or 2 PanNET with positive somatostatin receptor imaging (SRI), particularly in symptomatic or slowly progressive cases after use of somatostatin analog (SSA) therapy (4). For patients with symptomatic or rapidly progressing PanNET, alkylating chemotherapy should be considered prior to the use of PRRT, everolimus or sunitinib (5).

The recently published NETTER-2 trial evaluated the efficacy of PRRT in combination with octreotide LAR as first-line therapy for a sub-group of grade 2 (proliferation marker Ki-67 ≥10%) and grade 3 gastroenteropancreatic (GEP) NET. It demonstrated significant improvements in median progression-free survival (PFS), positioning it as a potential new standard of care in the first-line setting (2). However, since long-term follow-up is missing, the trial did not report on overall survival outcomes.

While the efficacy of PRRT was demonstrated in randomized controlled trials, real-world studies are essential to understand how these findings translate to broader, unselected patient populations (6, 7). This includes individuals with different tumor grades and functioning versus non-functioning tumors, which are often not fully represented in controlled trials (1, 2). This study addresses this gap by investigating the effects of PRRT on PanNET patients, focusing on the impact of tumor grading, functionality and other prognostic factors on survival outcomes. In addition, we focus on the real-world incidence of treatment discontinuation and its causes in a single center.

## Materials and methods

Patients were identified from our prospective NET database at the European Neuroendocrine Tumor Society (ENETS) Center of Excellence, Department of Endocrinology, Diabetes and Metabolism, University Hospital Essen, Germany. Eligible patients were those with histologically confirmed, differentiated, metastatic PanNET treated at our center between September 2010 and August 2024 with all records in our endocrine tumor center. All patients were reviewed by a multidisciplinary tumor board before initiation and at completion of treatment. Tumor response was assessed according to the Response Evaluation Criteria in Solid Tumors (RECIST) version 1.1.

Data is reported as the number of patients (percentage of group) for categories and as median (lower-upper quartiles) for quantitative variables, unless otherwise noted. Progression-free survival (PFS) was calculated from the first day of the first PRRT cycle to documented progression or death. Overall survival (OS) was measured from initial NET diagnosis to death from any cause. OS and PFS were analyzed using the Kaplan-Meier method and compared using the log-rank test. Bonferroni correction and False Discovery Rate (FDR) adjustment were applied to control for multiple comparisons and reduce the risk of Type I errors. To account for potential confounding factors, a multivariate Cox proportional hazards regression analysis was conducted to evaluate the independent effects of treatment cycles on OS and PFS. Tests were two-tailed and results at p < 0.05 were considered statistically significant. All statistical analyses were performed using R 4.4.2 (Posit Software, PBC, Boston, MA, USA). Written informed patient consent and approval for data collection and analysis were obtained upon admission to our institution. The study was approved by the Ethics Committee of the Medical Faculty of the University of Duisburg-Essen (18-8367-BO).

## Results

### Patient characteristics

We identified 166 consecutive metastatic PanNET patients, 82 females and 84 males (**Table 1**). The median age of the cohort at initial diagnosis was 56 years. The median Ki-67 index was 7%, with the Ki-67 data available for 161 patients (97%). Tumor grading revealed that 35 patients were classified as G1, 105 as G2 and 21 as G3 based on the World Health Organization criteria (8). Among the cohort, 19 patients (11.4%) had functioning tumors, including nine insulinomas (5.4%), eight gastrinomas (4.8%), and two VIPomas (1.2%), while the remaining 147 patients (88.6%) had non-functioning tumors. At the time of analysis, 100 patients (60.2%) were deceased.

**Table 1:** Patient characteristics of the whole PanNET cohort (n=166) and the PRRT PanNET cohort (n=100).

Characteristic	Whole Cohort	PRRT Cohort
**Total Number of Patients**	166 (100%)	100 (100%)
Sex		
**- Female**	82 (49.4%)	41 (41%)
**- Male**	84 (50.6%)	59 (59%)
Patient Status		
**- Alive**	66 (39.8%)	37 (37%)
**- Dead**	100 (60.2%)	63 (63%)
**Median Age at Initial Diagnosis, years**	56	56
Grading		
**- G1**	35 (21.1%)	21 (21%)
**- G2**	105 (63.3%)	68 (68%)
**- G3**	21 (12.7%)	9 (9%)
**- Unknown**	5 (2.9%)	2 (2%)
Ki-67 Index		
**- 0-5%**	76 (45.8%)	44 (44%)
**- 6-10%**	42 (25.3%)	27 (27%)
**- 11-20%**	22 (13.3%)	18 (18%)
**- >20%**	21 (12.7%)	9 (9%)
**- Unknown**	5 (2.9%)	2 (2%)
**Median Ki-67 Index, %**	7	8
NET Functionality		
**- Non-Functioning**	147 (88.6%)	88 (88%)
**- Functioning**	19 (11.4%)	12 (12%)
**Median Observation Time, months**	77	81
**Median Overall Survival, months**	79	83
**Patients with Bone Metastases**	60 (36.1%)	43 (43%)
**Median Cycles of PRRT**	–	4
**Number of PRRT Cycles**	–	
**- <4 cycles**	–	32 (32%)
**- 4 cycles**	–	45 (45%)
**- 5-8 cycles**	–	19 (19%)
**- >8 cycles**	–	4 (4%)

Bold values indicate headings.

### PRRT subgroup analysis

Within the cohort, 100 patients (60.2%) received PRRT. There were 41 females and 59 males in this subgroup. The median age at initial diagnosis remained consistent at 56 years. Tumor grading in the PRRT group showed 21 patients with G1, 68 with G2 and nine with G3 tumors and a median Ki-67 index of 8%. Tumor functionality in the PRRT group showed that 88 were non-functioning (88%) and 12 were functioning (12%) with seven insulinomas (7%), four gastrinomas (4%) and one VIPoma (1%). Patient status in this subgroup showed that 37 patients (37%) were alive and 63 were deceased (63%). The median number of cycles of PRRT administered was four. In patients who received less than four cycles of PRRT (n=17), the reason for PRRT discontinuation was progressive disease (n=8), hematotoxicity (n=2), increase in liver enzymes (n=1), inadequate SSTR uptake (n=1), duodenal perforation (n=1) and death (n=4). In two additional patients, PRRT was discontinued due to progressive disease after cycle five (n=1) and renal failure after cycle nine (n=1). In total, PRRT was discontinued in 19 patients (19%) due to adverse events.

### Progression-free survival and response rate

PFS was analyzed in patients who received four and eight continuous cycles of PRRT. Fifty patients had progressed after four cycles of PRRT, with a median PFS of 20 months (95% CI 15-25 months). The best responses after four cycles were partial response (PR) in 19 patients (38%) and stable disease in 17 patients (34%). Treatment failure was observed in 14 patients, with progressive disease (PD) in 12 patients (24%) and mixed response in two patients (4%), leading to a change in treatment. The disease control rate (DCR) was 72% and the objective response rate (ORR) was 38%.

Twelve patients who completed eight cycles of PRRT were evaluated with a median PFS of 18 months (95% CI 10-26 months). The difference in PFS was not statistically significant (p = 0.4). The best responses included PR in three patients (25%), stable disease in six patients (50%) and PD in three patients (25%). The DCR was 75% and the ORR was 25%.

### Overall survival

**Figure 1:**
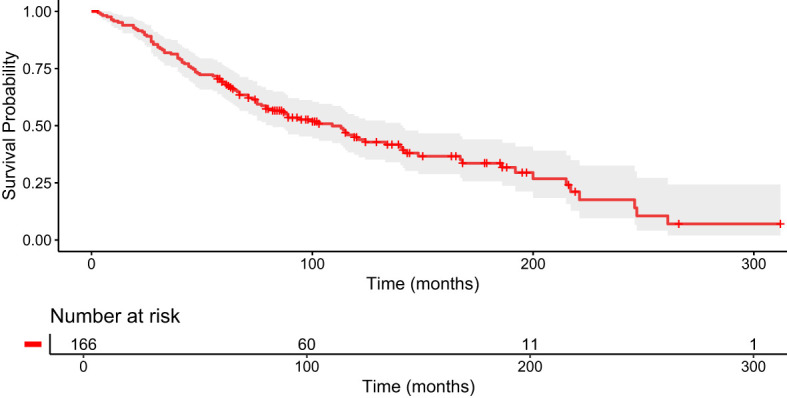
Kaplan–Meier curve for median OS in months for the entire cohort, n=166.

The overall median OS for the entire cohort of 166 patients was 79 months (95% CI 7-251), with a median Ki-67 index of 7% (**Figure 1**). Patients who did not receive PRRT had a lower median OS of 67 months (95% CI 4-234), with a median Ki-67 index of 5% (**Table 2**). The subgroup of patients who received exactly two cycles of PRRT had the lowest median OS at 59 months (95% CI 21-210). This group had a higher median Ki-67 index of 10% and a significant number of patients with bone metastases (60%).

**Table 2:** Median OS and patient characteristics for the whole cohort and subgroups.

Group, n	Median OS in months	Median Ki-67 in %	Median Age at initial diagnosis	Patients with bone metastases, n	Sex female/male
**Whole cohort (n=166)**	79	7	56	36% (60)	82/84
**No PRRT (n= 66)**	67	5	56	26% (17)	41/25
**2 cycles PRRT (n=15)**	59	10	52	60% (9)	8/7
**4 cycles PRRT (n=45)**	79	5	56	42% (19)	19/26
**4+ cycles PRRT (n=68)**	87	7	56	37% (25)	27/41
**5+ cycles PRRT (n=23)**	100	10	55	26% (6)	8/15

Bold values indicate headings.

**Figure 2:**
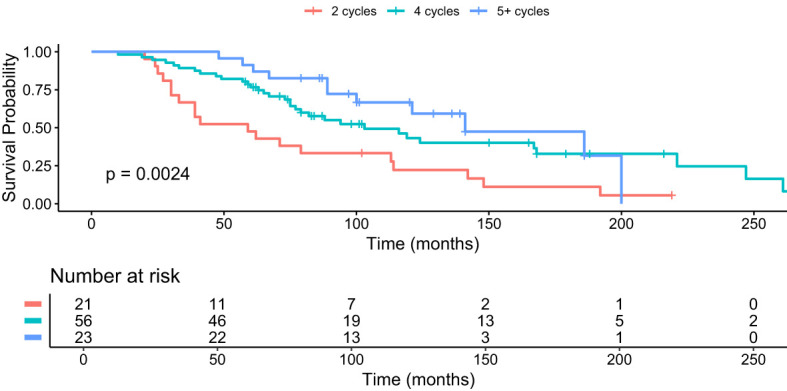
Kaplan–Meier analysis for median OS in months for subgroups who received 2 cycles (red), 4 cycles (green) and 5+ cycles (blue) of PRRT, p = 0.0024.

Patients who received four cycles of PRRT had a median OS of 79 months (95% CI 19-223), the same as the overall cohort (**Figure 2**). This subgroup had a lower median Ki-67 index of 5%, a median age at diagnosis of 56 years and 42% of patients with bone metastases. There was a slight male predominance compared to those who did not receive PRRT.

Patients who received four cycles and more of PRRT (“4+ cycles”) showed a median OS of 87 months (95% CI 22-220) and a median Ki-67 index of 10%. The median age at diagnosis was 56 years, 37% had bone metastases and 60% were male.

Patients who received 5+ cycles PRRT showed the longest median OS of 100 months (95% CI 52-194) despite a higher median Ki-67 index of 10%. The proportion of patients with bone metastases was lower at 26%, comparable to the “no PRRT” group. The median age at diagnosis was 55 years, the youngest of all subgroups, with a male predominance of 65%.

### Grading, functionality and bone metastases

**Figure 3:**
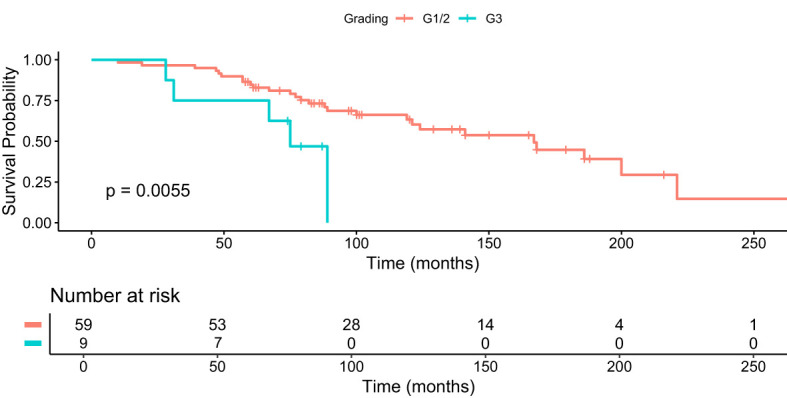
Kaplan–Meier analysis for median OS in months for patients with G1/2 and G3 who received 4+ cycles of PRRT.

We further analyzed the median OS in patients who received 4+ cycles of PRRT, i.e. the largest group with a longer median survival in correlation to different gradings. The median OS was 97 months (95% CI 84-101) in G1 and G2 PanNET patients and 74.5 months (95% CI 31-79) in G3 PanNET patients (**Figure 3**). The difference was statistically significant (p = 0.0055, Bonferroni-adjusted p = 0.022, FDR-adjusted p = 0.011).

When comparing the non-functioning NET group to the functioning group, the median overall survival was 88.5 months (95% 79-100) vs 81 months (95% CI 61-87) (p = 0.82) when treated with 4+ cycles of PRRT.

**Figure 4:**
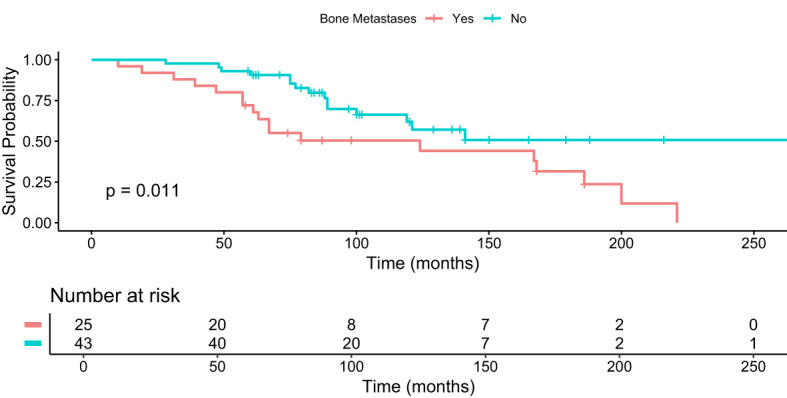
Kaplan–Meier analysis for median OS in months for patients with and without bone metastases who received 4+ cycles of PRRT.

Patients with bone metastases who received 4+ cycles of PRRT had a median OS of 74 months (95% 61-87) compared to 89 months (95% CI 84-101) in patients who did not have bone metastases (**Figure 4**). The difference was statistically significant (p = 0.013, FDR adjustment (p = 0.017), Bonferroni correction (p = 0.052)).

### Multivariate analysis

**Figure 5:**
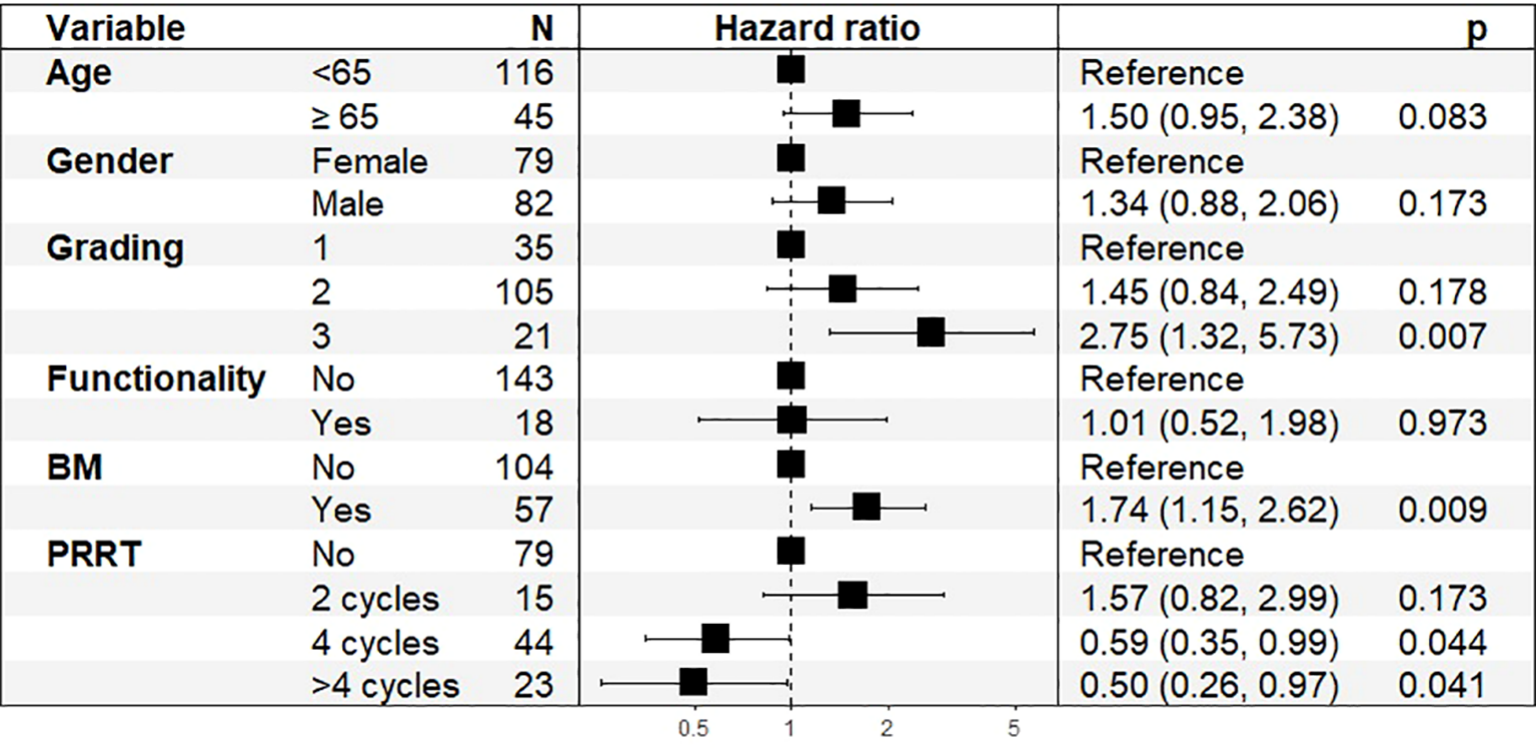
Hazard Ratios (HR) and 95% Confidence Intervals (CI) for Overall Survival in total cohort, n=161.

We conducted a Cox proportional hazards regression analysis to evaluate the prognostic impact of various clinical parameters on OS and PFS after 4 cycles of PRRT. In the total cohort, the multivariate Cox regression analysis identified significant predictors of OS (**Figure 5**).

Higher tumor grading was a significant prognostic factor (HR: 2.75, 95% CI: 1.32–5.73, p = 0.007), with G3 tumors showing worse survival outcomes. The presence of bone metastases was significantly associated with poorer survival (HR: 1.74, 95% CI: 1.15–2.62, p = 0.009). PRRT treatment showed a significant association with improved OS, with HRs of 0.59 (95% CI: 0.35–0.99, p = 0.044) for 4 cycles and 0.50 (95% CI: 0.26–0.97, p = 0.041) for more than 4 cycles. Age at diagnosis showed a trend toward statistical significance, with an estimated hazard ratio (HR) of 1.50 (95% CI: 0.95–2.38, p = 0.083), indicating a possible increase in mortality risk with advancing age.

**Figure 6:**
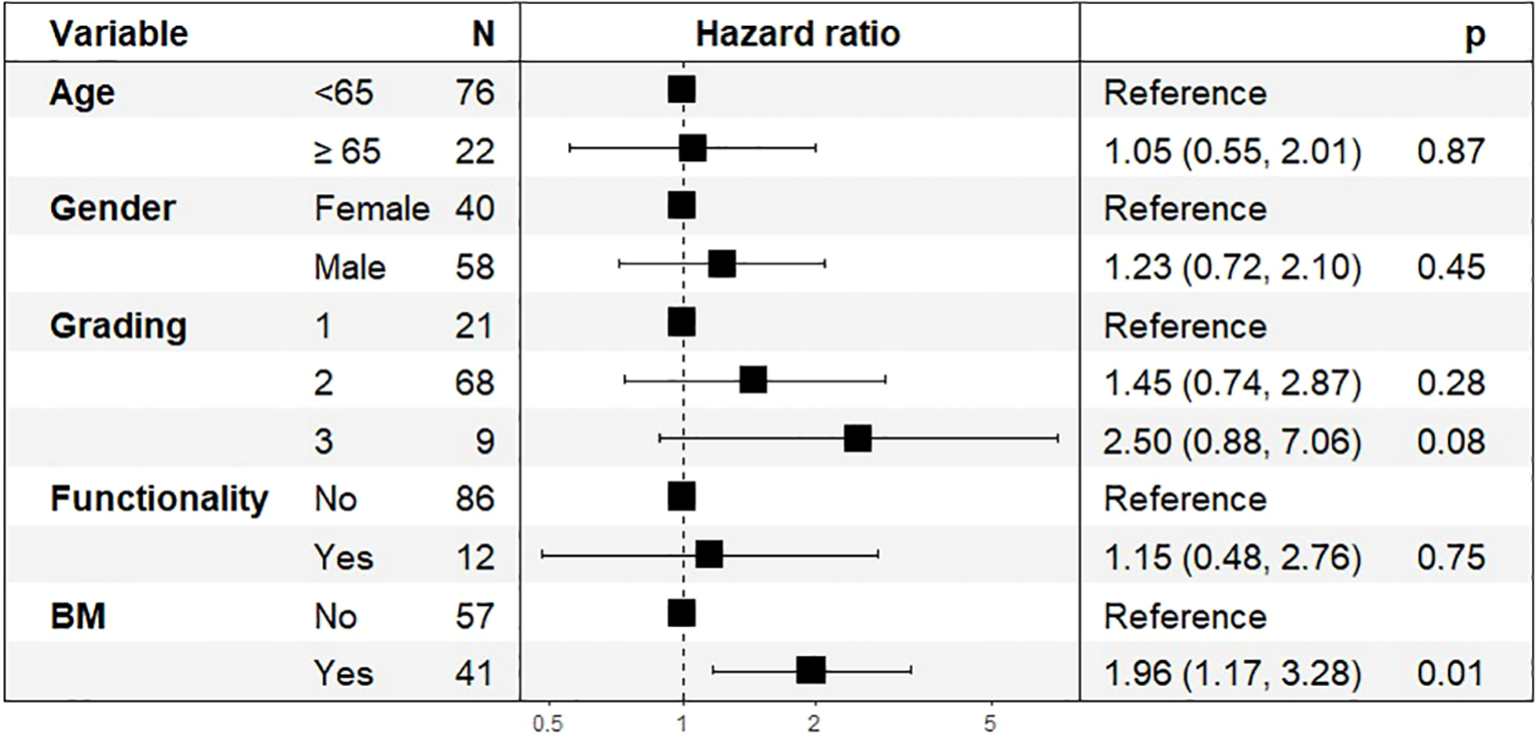
Hazard Ratios (HR) and 95% Confidence Intervals (CI) for Overall Survival in PRRT cohort, n=98.

In the subgroup of patients who received PRRT (**Figure 6**), the Cox regression model showed that bone metastases remained a significant prognostic factor for worse survival (HR: 1.96, 95% CI: 1.17–3.28, p = 0.01). Tumor grading (HR: 2.50, 95% CI: 0.88–7.06, p = 0.08) and age (HR: 1.05, 95% CI: 0.55–2.01, p = 0.87) were not statistically significant in this subgroup. Gender and tumor functionality did not show significant associations with OS in the PRRT subgroup.

**Figure 7:**
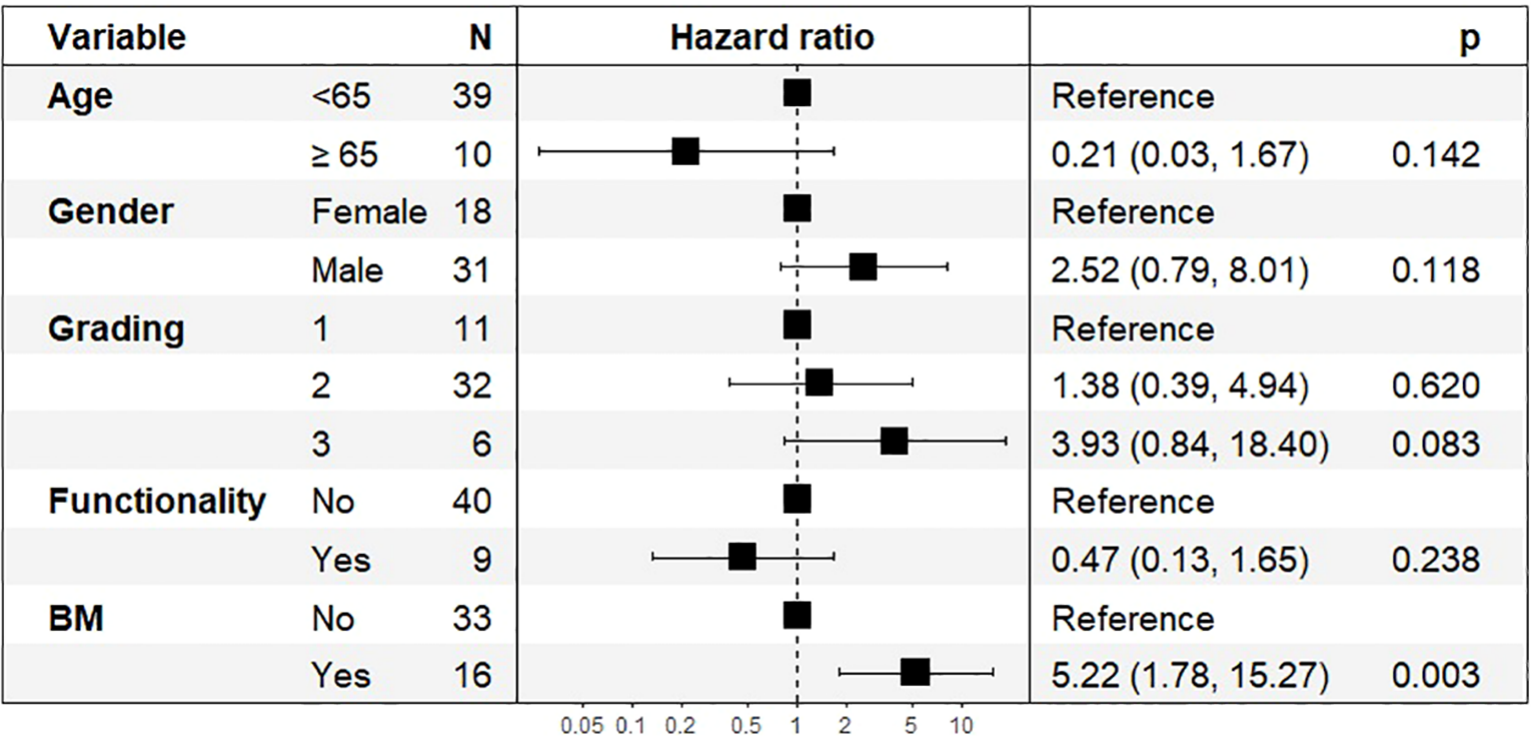
Hazard Ratios (HR) and 95% Confidence Intervals (CI) for Progression-Free Survival after 4 PRRT Cycles, n=49.

PFS after four continuous PRRT cycles was analyzed in 49 patients (**Figure 7**). The presence of bone metastases was the strongest predictor of shorter PFS, with an HR of 5.57 (95% CI: 1.73–17.87, p = 0.004). Tumor grading (HR: 4.03, 95% CI: 0.85–19.10, p = 0.079) did not reach statistical significance. Age at diagnosis, tumor functionality and gender were not significant predictors of PFS in this cohort.

## Discussion

Our study analyzed the impact of grading and functionality on the effectiveness of PRRT in patients with metastatic PanNET. Approximately 60% of our PanNET cohort underwent PRRT, highlighting the frequent use of PRRT at our center. The high mortality rate in our cohort (60.2% deceased at the time of analysis) underscores the aggressive nature of metastatic PanNET and the need for optimized treatment strategies (4, 9–11).

PRRT demonstrated favorable disease control in patients with advanced PanNET, achieving a DCR of 72% after four cycles and 75% after eight cycles. The ORR reflecting the proportion of patients who achieved PR was 38% after four cycles and decreased to 25% after eight cycles. This suggests that while PRRT continues to stabilize disease in most patients throughout treatment, the likelihood of achieving a significant tumor shrinkage diminishes as treatment progresses. The DCR remained high, indicating that PRRT effectively halts tumor progression in most patients, even with extended cycles.

Recently, the authors of the NETTER-2 data stated that PRRT should be considered a new standard of care as first-line therapy for grade 2 and 3 GEP-NET, extending median PFS to 22.8 months in the PRRT arm compared to 8.5 months in the high-dose octreotide 60 mg LAR (control) arm (2).

In contrast, our study analyzed a cohort of metastatic PanNET patients, including those who received different cycles of PRRT. Compared to NETTER-2, which focused on first-line therapy, our study includes any-line therapy. This distinction is critical as our results reflect outcomes across different phases of treatment rather than a first-line intervention. NETTER-2’s focus on G2/3 tumors contrasts with our inclusion of more than 20% of G1 PanNET, allowing for a more comprehensive evaluation of the real-world effectiveness of PRRT. This likely reflects the common practice of using PRRT not only in advanced, high-grade tumors but also in well-differentiated, lower-grade PanNET underscoring the potential role of PRRT beyond its traditional indication for more aggressive disease. The current developments might lead to an even more prominent role of PRRT in the treatment of low-grade PanNET in the future.

The OCLURANDOM trial is the first multicenter, randomized, open-label phase II study to evaluate the anti-tumor activity of Lu-177 DOTATATE (12). The two-arm randomized study of PRRT and sunitinib met its primary endpoint by achieving significant PFS with a median of 20.7 months in the PRRT arm and 11 months in the sunitinib arm. Median PFS in our PRRT cohort was similar with 20 and 18 months at four and eight cycles, respectively. Other trials evaluating the efficacy of PRRT are ongoing, such as COMPOSE (13).

Another important finding of our study is the association between the number of PRRT cycles and OS. Patients who received more than four cycles of PRRT showed the longest median OS of 100 months, despite a higher median Ki-67 index of 10%, which may be related to earlier and more aggressive interventions. Multivariate Cox regression analysis showed a significant association with improved OS in the PRRT cohort, with HRs of 0.59 (95% CI: 0.35–0.99, p = 0.044) for 4 cycles and 0.50 (95% CI: 0.26–0.97, p = 0.041) for more than four cycles.

Our results also highlight the significant heterogeneity in patient response to PRRT. For example, the cohort of patients who received exactly two cycles of PRRT had a significantly lower median OS of 59 months, suggesting a limited benefit from fewer cycles. The presence of bone metastases had a significant impact on OS in our study. Patients with bone metastases who received 4+ cycles of PRRT had a median OS of 74 months compared to 89 months for patients without bone metastases (p = 0.013). This finding highlights the challenge of treating bone metastases and suggests that additional or more aggressive therapeutic strategies may be needed for patients with such metastases (14). It was further supported by our multivariate analysis, where bone metastases remained a significant prognostic factor in the PRRT subgroup (HR: 1.96, p = 0.01).

In contrast, patients with lower-grade tumors (G1/G2) and lower Ki-67 indices who received 4+ cycles of PRRT had a significantly longer median OS of 87 months vs. 74.5 months in G3 PanNET.

Interestingly, no significant difference in OS was observed between patients with functioning versus non-functioning PanNET who received 4+ cycles of PRRT (median OS of 81 months vs. 88.5 months, p = 0.82). This suggests that the functional status of the tumor may not significantly influence the response to PRRT. This finding may support the broader use of PRRT across different PanNET subtypes, regardless of their hormonal activity.

Notably, PRRT was discontinued in 19% of patients due to progressive disease, toxicity or death, highlighting the importance of monitoring treatment tolerability. The longevity of toxic effects remains to be analyzed and long-term follow-up is required.

Selection bias, particularly due to survival bias, may have influenced our results. Patients who lived long enough to receive multiple cycles of PRRT likely had inherently better overall health, characterized by favorable prognostic factors such as robust blood counts, preserved renal function and SRI positivity. These factors do not only make patients eligible for PRRT, they are also independently associated with improved survival, which may partly explain the longer OS observed in those who received four or more cycles. The lack of a randomized control or prospective study design further complicates the interpretation of the survival benefit of PRRT, as patients with more favorable baseline characteristics may have been preferentially selected to continue therapy.

A major limitation of the study is that treatment sequencing was not analyzed. The heterogeneity of patient presentation, tumor biology and response to treatment made it difficult to establish comparable subgroups for analysis of treatment sequencing. As a result, we could not determine the optimal sequence or combination of treatments, such as PRRT, chemotherapy or molecular targeted therapies, which limits the ability to provide clear guidance for clinical practice. The diversity of tumor grades, Ki-67 indices, and progression patterns make it difficult to draw generalized conclusions about the best treatment pathways for metastatic PanNET. Future prospective studies are needed to explore optimal sequencing strategies to improve treatment efficacy and patient outcomes.

A key area for future research is to determine which patients are most likely to benefit from re-PRRT and the optimal timing for re-treatment. Further studies should investigate whether patients with more aggressive tumors (higher Ki-67) could benefit from earlier initiation of PRRT and whether subsequent re-treatment at specific intervals could further prolong survival. The optimal interval between PRRT courses remains an open question and could be influenced by factors such as tumor biology, previous treatment response and overall patient condition.

In conclusion, while our study confirms the benefit of PRRT in prolonging survival in metastatic PanNET patients, particularly with more cycles, it also highlights the need for comparative studies and further investigation into treatment sequencing and personalized patient management. Integrating findings from studies such as NETTER-2 with our results may provide a more nuanced understanding of the role of PRRT in treating advanced PanNET and improving patient outcomes.

## Conclusion

PRRT demonstrated prolonged disease stabilization and a potential survival benefit in metastatic PanNET patients, especially when administered in extended cycles. G3 PanNET and bone metastases remain challenging, highlighting the need for tailored therapeutic strategies in this subset. These findings align with the results of randomized trials such as NETTER-2, while further highlighting the prolonged overall survival benefit, underscoring the role of PRRT as a valuable treatment option including functioning PanNET. Further research is needed to optimize the sequencing of therapy, understand the role and timing of re-PRRT and explore personalized approaches that integrate PRRT with other systemic treatments to improve patient outcomes.

## Data Availability

The raw data supporting the conclusions of this article will be made available by the authors, without undue reservation.
